# The role of tumor budding in the prognosis of patient with locally advanced colorectal cancer

**DOI:** 10.3389/fonc.2026.1808095

**Published:** 2026-03-25

**Authors:** Muzaffer Çaydere, Fatma Aslan Yay, Zümrüt Merve Yaşaran Benk, Yağmur Çiman, İbrahim Emre Bulut

**Affiliations:** 1 Department of Pathology, University of HealthSciences, Ankara Training and Research Hospital, Istanbul, Türkiye; 2 Ankara Training and Research Hospital, Istanbul, Türkiye; 3 Sincan Training and Research Hospital, Istanbul, Türkiye

**Keywords:** colorectal carcinoma, locally advance, prognostic factors, stage II and III, tumor budding

## Abstract

Research on the use of tumor budding (BD), an important survival marker for colorectal carcinoma (CRC), is still ongoing. In the present study, we aimed to determine the effectiveness of BD on prognosis in locally advanced CRC cases. In our study, CRC cases operated on between 2005 and 2015 were examined.BD was calculated using a standard methodology. A significant relationship was found between poor prognostic factors and BD (lymphatic invasion [p=0.008], tumor perforation [p=0.023], locally advanced-stage [p=0.023], positive surgical margins [p=0.005], advanced pT [p<0.001], and high grade [p<0.001]). Additionally, we found poor prognostic factors to be an independent risk factor in the regression analysis for BD (OS = 2.85 [1.15-3.48], p=0.001). Furthermore, correlation (r=0.695) and repeatability analysis (Kappa=0.56-0.75) for BD data indicated that it was successful (ROC: 10.23; AUC = 0.822 [0.714-0.943]). There was a significant difference in survival on univariate analysis for BD (RFS<0.001, OS = 0.001). In multivariate analysis, BD was shown to be an independent poor prognostic factor for CRC (RFS: HR = 2.96 [1.48-3.57], p<0001), and OS: HR = 2.69 [1.38-3.70], p<0.001). Our study indicates that BD detected using a standard methodology is a suitable predictive factor in determining poor prognosis in locally advanced CRC cases.

## 1 Introduction

Colorectal cancer (CRC) is a very common tumor and the third most common tumor worldwide. However, the majority of cases are diagnosed late (1). Many large-scale studies on colorectal cancer have been conducted in the literature, and significant advances have been made in prognostic and predictive factors (2). Today, the TNM staging system is widely used to predict prognosis globally (3). Unfortunately, even employing this method, some clinical processes are difficult to predict. For example, significant differences in clinical behavior can be observed between patients at the same stage, and there is no consensus on which patients should receive neoadjuvant chemotherapy (3, 4). Alas, existing classifications cannot provide sufficient assistance in selecting the ideal patient in this regard, and there continues to be a need for studies on new prognostic markers (5).

Tumor budding (BD) can be briefly defined as the presence of small tumor cell foci (<=5 cells) or individual tumor cells at the invasive edge of the tumor (6). BD is thought to play an important role in the transition to mesenchyme in the invasive region of the tumor and is the first indicator of local and distant metastasis and invasion of surrounding organs (6, 7). Many studies have shown that increasing the number of tumor buds is associated with an increased rate of recurrence and metastasis in CRC (8–15). Although it is believed that the inclusion of tumor budding in the list of high-risk factors in CRC is justified, a lack of standardization exists on how to report BD. Additionally, there are only a few studies evaluating BD by immunohistochemistry (8–15).

In this study, we aimed to identify at-risk individuals in locally advanced CRC patients using a standard method. This research stands out by using a very homogeneous sample and a standard methodology.

## 2 Materials and methods

**Figure 1:**
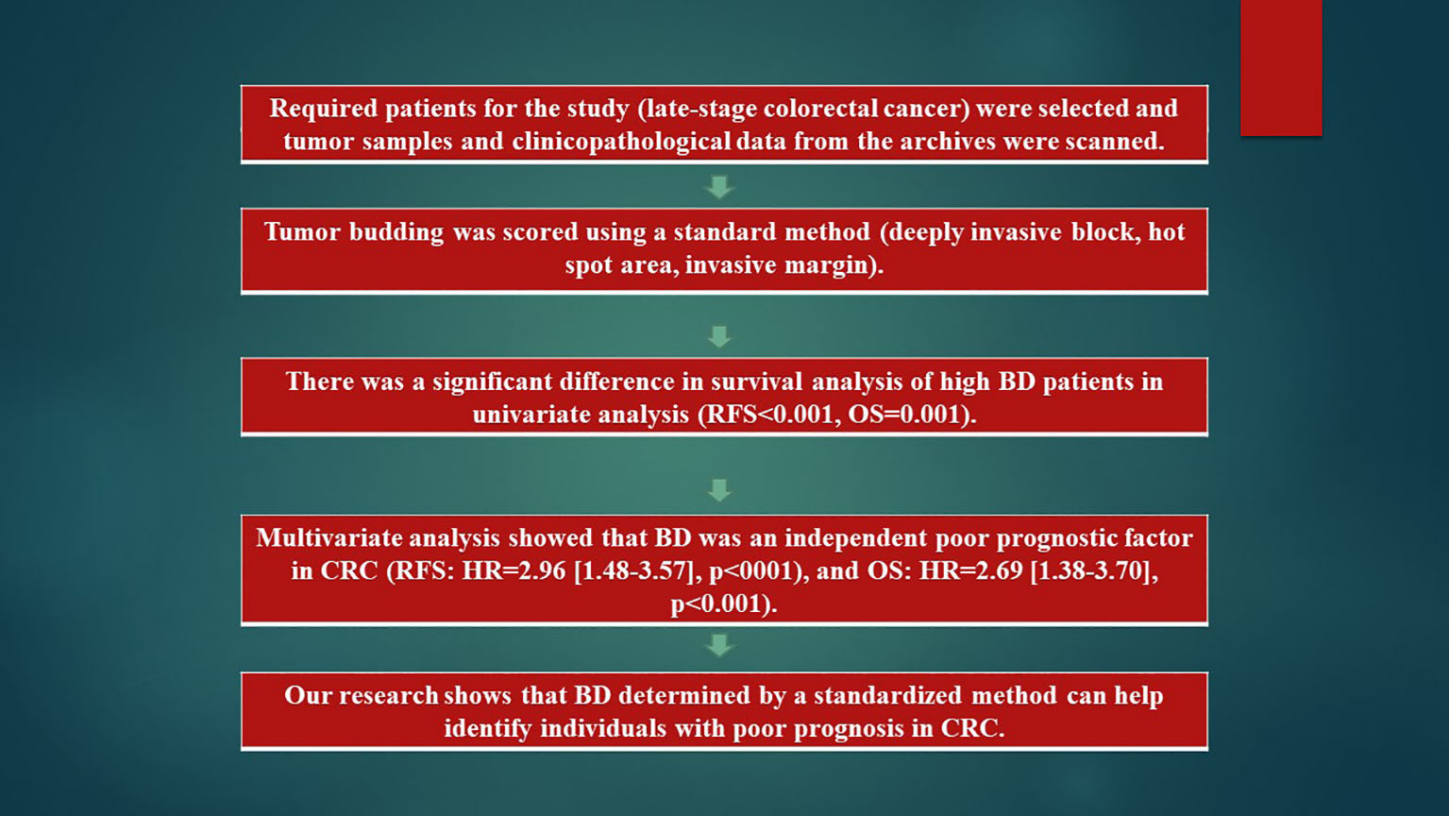
Flowchart. CRC, Colorectal cancer; H&E, Hematoxylin and eosin; HR, Hazard ratio; OS, Overall survival; RFS, Relapse-free survival.

This study is briefly summarized in **Figure 1** and was carried out using REMARK (16) rules.

### 2.1 Ethical standards

Approval for this research was received from Ankara Training and Research Hospital Ethics Committee. It was also conducted in accordance with all national ethical norms and the Declaration of Helsinki protocol. The study obtained written informed consent from each patient participating in the study.

### 2.2 Research design

Our research was carried out in the Pathology Department of Ankara Training and Research Hospital. While generating the population for this study, patients (n=364) who underwent CRC surgery between 2005 and 2015 were scanned.

### 2.3 Patient selection

Patients with distant metastases (n=9), those with more than one malignancy simultaneously (n=5), those who died within the first month, or those who survived recurrence were not included in the study (n=8). Other exclusion criteria included those with no tumor found in the archives (n=11), those previously diagnosed with cancer (n=5), those without sufficient tissue for evaluation (n=5), those with early tumor stages (n=51), and those treated with adjuvant recovery/radiotherapy (n=10). After extracting these patients from the study, our population consisted of 260 samples.

### 2.4 Tissue

The examined tissue tumor samples were taken from the archives of the Department of Pathology. Block numbers ranged from 3 to 42 per case. A tumor block representing the most invasive area was used for each patient. Care was taken to include both tumor and normal colon tissue in the block we selected for immunohistochemistry (IHC) analysis. Two sections (four microns) were taken from the blocks we selected. One of these sections was stained with routinely used hematoxylin and eosin (H&E), and the other was stained with pan-cytokeratin (AE1/AE3). The 7th edition of AJCC was considered in all pathological evaluations (17). Two pathologists evaluated each piece, and the final BD was calculated as the average of these two evaluations.

### 2.5 Evaluation for BD

When we review the literature, we see that BD has been evaluated using a wide variety of methodologies (8–15). We evaluated BD in the deepest invasive block, at the invasive edge of the tumor, and in the “hot-spot” area. We considered a bud to be a small carcinoma cluster consisting of up to four tumor cells or a solitary isolated adenocarcinoma cell (18, 19). We counted the number of tumor buds in H&E and IHK-stained preparations using a standard light microscope (0.40 mm, 1.256 mm², Nikon Eclipse E600, Switzerland).

**Figure 2:**
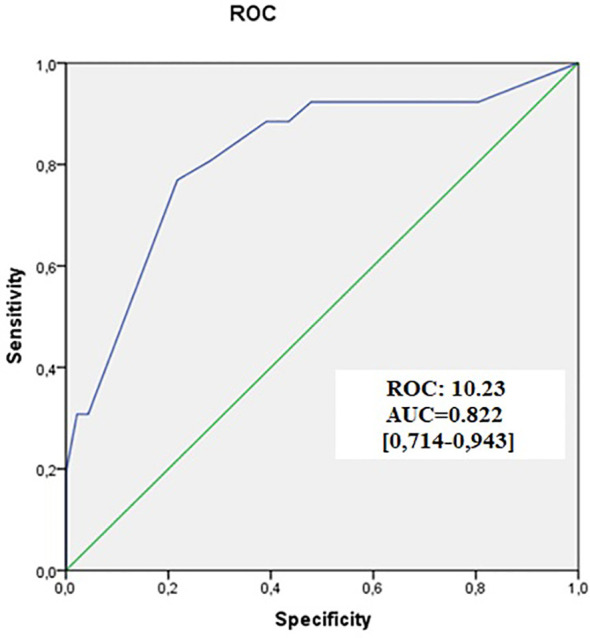
BD calculation. First, we scanned all sections using an x10 objective. Then we chose an area suitable for the above-mentioned methodology. Finally, we counted using an x20 objective.

First, we scanned all available preparations using a 10x objective to find the BD concentration. Next, we selected an area suitable for the method mentioned above and counted with a20x objective (**Figure 2**). When counting tumor cells, an attempt was made to exclude cytoplasmic fragments and artefacts as much as possible.

### 2.6 Cut-off value

We know that if we do not determine the cut-off value correctly in our research, it will not be possible to achieve successful results. The true threshold value is the highest true positive rate and the lowest false positive rate. We investigated this value using a ROC test. We grouped all our cases according to this value (low-high intensity). Additionally, the larger the area under the ROC curve (AUC), the more effective the test. In this way, we also analyzed the usefulness of the test.

### 2.7 Reproducibility

Tumor heterogeneity and interobserver agreement were analyzed to assess reproducibility. An intraclass correlation coefficient (ICC) test was used to evaluate tumor heterogeneity (20). The ICC was defined as the ratio of the total variance among the tumors examined. If this ratio were due to biological diversity or intratumor variation, the ICC would be close to one; if it were due to heterogeneity within the tumor, it would be close to zero. We used the Kappa(K) test to evaluate interobserver agreement. According to Landis et al. (21), a classified K value between 0.81–1 is considered excellent, between 0.61-0.80 is moderate, and between 0.41-0.60 is significant.

### 2.8 Follow-up

Our study focused on survival and relapse rates for follow-up and determined a follow-up period of at least five years for each case. The time from the day of surgery to the occurrence of relapse was used to define relapse-free survival (RFS). The time from the date of surgery until the last follow-up or the patient’s death was defined as overall survival (OS). Relapse refers to the clinical, radiological, and pathological reappearance of the tumor. Relapse occurring in the treated area was defined as local relapse, and relapse spreading to another region was defined as distant relapse.

### 2.9 Immunohistochemistry

We took a 4 μm section onto a platinum-coated slide for IHC analysis. We recovered the retrieval epitope by heating a targeting solution at pH9 and 97 °C.A peroxidase-blocking agent (Dako) was used to inhibit endogenous peroxidase activity. We chose monoclonal AE1/AE3 as the primary antibody (Dako, 1:100, M3010, Mouse) and also used MLH1 (Dako), MSH2 (Dako), MSH6 (Dako), and PMS2 to assess microsatellite instability. We kept these antibodies at room temperature for incubation (30 minutes). DAB and HRP reactions were used to visualize incubated antibodies. After applying hematoxylin (Merck) as a counterstain, we covered the slides with a coverslip.

### 2.10 Statistical analysis

We used the SPSS 24.0 program (North Castle, USA) for all analyses. Standard deviation, mean, range, frequency, and percentage values ​​were used as descriptive variables. We preferred the chi-square test to examine the statistical relationship between clinicopathological parameters and BD. The Spearman correlation test and the Wilcoxon signed-rank test were employed to examine correlations and differences in our continuous data. ROC analysis determined the ideal threshold value of BD. The log-rank test evaluate differences between univariate survival groups. We used the Kaplan-Meier technique when presenting survival curves. When we analyzed the differences between the survival group multivariable, we used the Cox regression test, and the ICC test to assess the variability of tumors. The K test was used to evaluate interobserver correlation. In all these analyses, the pvalue was considered​​to be significant when it was below 0.05.

## 3 Results

### 3.1 General features

The average age in the present study was 68.56 ± 9.52 (range: 38-88), and the tumor size was 6.32 ± 1.56 (range: 2-13). Gender distribution of the cases revealed that 157 (60.3%) were men and 103 (39.7%) were women. When we examined the degree of differentiation of the cases, 96 (36.9%) were poorly differentiated, and 164 (63.1%) were well/medium differentiated. The number of patients with pT4 was 104 (40.0%) and the number of patients with pT3 was 156 (60.0%).

### 3.2 BD analysis

We evaluated BD in H&E and immunostained sections using the “deepest invasive block, hot spot area, invasive margin”method. When we screened the preparations, we determined that the distribution of BDs was heterogeneous. The median number of BDs was 8.4 and the mean number was 9.32 ± 3.48 (range: 0-25). When we analyzed the BD numbers separately, we found that they ranged from 4.6-13.4 for H&E sections and 5.1-16.3 for immune sections. Representative images of our analysis are shown in **Figure 2**.

### 3.3 Relationship of BD with clinicopathological features

**Figure 3:**
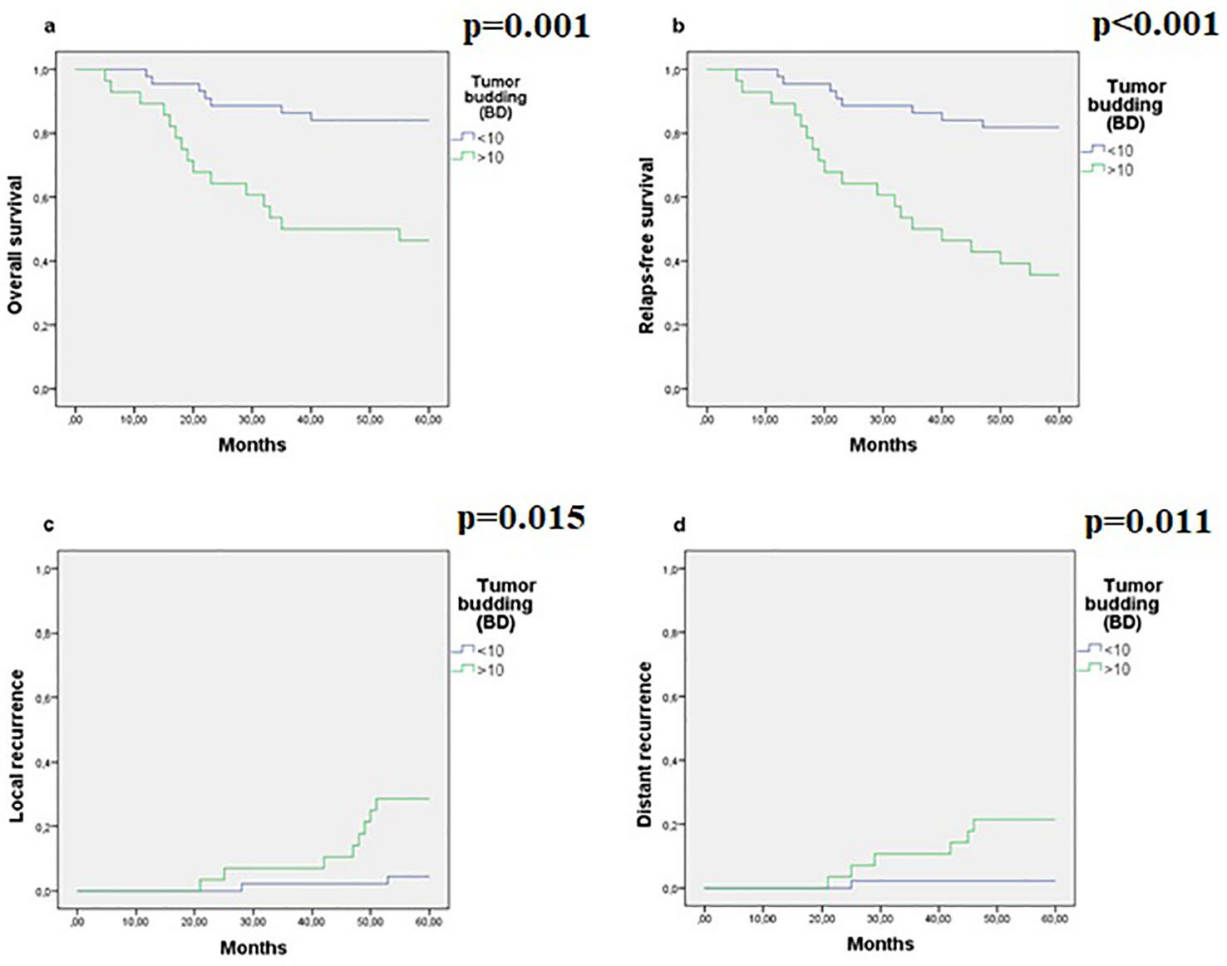
ROC analysis. The optimum cut-off value for BD was analyzed usingthe ROC test. BD, Tumor budding; ROC, Receiver Operating Characteristic; AUC, Areas under the ROC curves. **(A)** Overall survival, **(B)** Relapse-free survival, **(C)** Local recurrence, **(D)** Distant recurrence.

In our results, we found that BD was associated with poor prognostic factors [lymphatic invasion [p=0.008], tumor perforation [p=0.023], locally advanced-stage [p=0.023], positive surgical margins [p=0.005], advanced pT [p<0.001], and high grade [p<0.001]] (**Table 1**). The logistic regression analysis indicated that BD was an independent parameter for CRC (OS = 2.85 [1.15-3.48], p = 0.001) (**Table 2**). The correlation and difference between the current method and BD estimates were significant (r=0.657-0.695; p<0.001) (**Table 3**). Additionally, we analyzed the optimal cut-off value (10.23) for BD (AUC = 0.822 [0.714-0.943]) (**Figure 3**). For ease of application, we accepted this value as ten and classified all cases accordingly.

**Table 1:** Statistical relationship between BD and prognostic factors in CRC.

Prognostic factors		BD (H&E)			BD (immun)		
		Positive	Negative	P-value	Positive	Negative	P-value
Age				0.680			0.721
	**<68**	48 (39%)	56 (40%)		45 (39%)	59 (40%)	
	**≥68**	74 (61%)	82 (60%)		69 (61%)	87 (60%)	
Gender				0.423			0.311
	**Female**	50 (41%)	53 (37%)		47 (41%)	56 (33%)	
	**Male**	72 (59%)	85 (63%)		67 (59%)	90 (63%)	
Size				0.555			0.353
	**<6.3 cm**	42 (35%)	57 (41%)		38 (36%)	61 (41%)	
	**≥6.3 cm**	80 (65%)	81 (59%)		76 (64%)	85 (59%)	
Localization				0.325			0.532
	**Right**	51 (42%)	53 (37%)		46 (4%)	58 (39%)	
	**Left**	71 (58%)	85 (63%)		68 (59%)	88 (61%)	
Perineural invasion				0.744			0.653
	**No**	48 (39%)	57 (41%)		45 (40%)	60 (41%)	
	**Yes**	74 (61%)	81 (59%)		69 (60%)	86 (59%)	
Lymphatic invasion				*0.011* ^*^			*0.008* ^*^
	**No**	58 (48%)	48 (34%)		55 (49%)	51 (44%)	
	**Yes**	64 (52%)	90 (66%)		59 (51%)	95 (56%)	
Surgical margin				*0.008* ^*^			0.005*
	**Negative**	59 (48%)	48 (34%)		56 (49%)	50 (33%)	
	**Positive**	63 (52%)	90 (66%)		58 (51%)	96 (67%)	
pT				*0.008* ^*^			<0.001^*^
	**pT IV**	60 (49%)	44 (31%)		58 (51%)	46 (31%)	
	**pT III**	62 (51%)	94 (69%)		56 (49%)	100 (69%)	
Grade				<0.001^*^			<0.001*
	**High**	56 (46%)	40 (28%)		53 (48%)	42 (28%)	
	**Low**	66 (54%)	98 (72%)		61 (52%)	104 (72%)	
Tumor perforation				*0.023* ^*^			0.021*
	**Negative**	60 (50%)	47 (33%)		57 (50%)	50 (33%)	
	**Positive**	60 (50%)	91 (67%)		57 (50%)	96 (67%)	
MSI Status				0.644			0.570
	**MMR-P**	52 (43%)	63 (45%)		43 (38%)	52 (35%)	
	**MMR-D**	70 (57%)	75 (55%)		71 (62%)	74 (65%)	

*P values ​​of 0.05 and below are significant and are shown in italics.

BD, Tumor budding; MSI, Microsatellite instability; MSS, Microsatellite stable; pT, Pathologic tumor stage; MMR-P, Mismatch Repair-Proficient; MMR-D, Mismatch Repair-Deficient.

**Table 2:** Logistic regression analysis.

Prognostic factors	Univariable		Multivariable	
	OR (95% CI)	P value	OR (95% CI)	P value
Lymphatic invasion	1.67 (0.72-4.11)	0.153	2.45 (0.88-4.23)	0.285
Surgical Margin	1.35 (1.57-3.77)	0.003*	1.77 (1.82-4.53)	0.005*
Stage	2.83 (0.94-4.77)	0.191	2.74 (0.85-3.63)	0.203
pT	2.32 (1.22-3.55)	0.031*	2.52 (1.25-3.33)	0.015*
Grade	1.22 (1.11-2.54)	0.001*	1.13 (1.35-2.46)	0.005*
Tumor perforation	2.55 (0.77-7.51)	0.152	2.77 (0.89-7.35)	0.065
BD	2.24 (1.12-3.48)	0.001*	2.85 (1.15-3.48)	0.001*

*P values ​​of 0.05 and below are significant and are shown in italics.

BD, Tumor budding; pT, Pathologic tumor stage.

**Table 3:** Correlation and reproducibility of BD.

Tumor budding	Correlation	Difference	ICC (95% CI)	Kappa values
BD (H&E)	0.657,p<0.001	0.335, p<0.001	0.645 (0.553-0.759)	0.56-0.71
BD (Immun)	0.695, p<0.001	0.326, p<0.001	0.677 (0.546-0.723)	0.58-0.75

*P values ​​of 0.05 and below are significant.

BD, Tumor budding; ICC, Intra-Class Correlation Coefficient.

### 3.4 Reproducibility results

We used the method described above when examining BD. Here, we only gave the best results and noted the continuous and categorical variables separately when noting the data. When we analyzed the interobserver agreement, we found that it was in the range of clinically significant (K=0.56–0.75). When we examined the K values, we saw that the IHC results were higher (**Table 3**). The heterogeneity of the tumors showed the IHC results were higher. When we examined the ICC values, for example, a value of 0.677 showed that 32.3% of the total variability was due to heterogeneity within the tumor. In other words, we determined that the intratumor variation was lower than the intertumor variation in our study (**Table 3**).

### 3.5 Follow-up results

**Figure 4:**
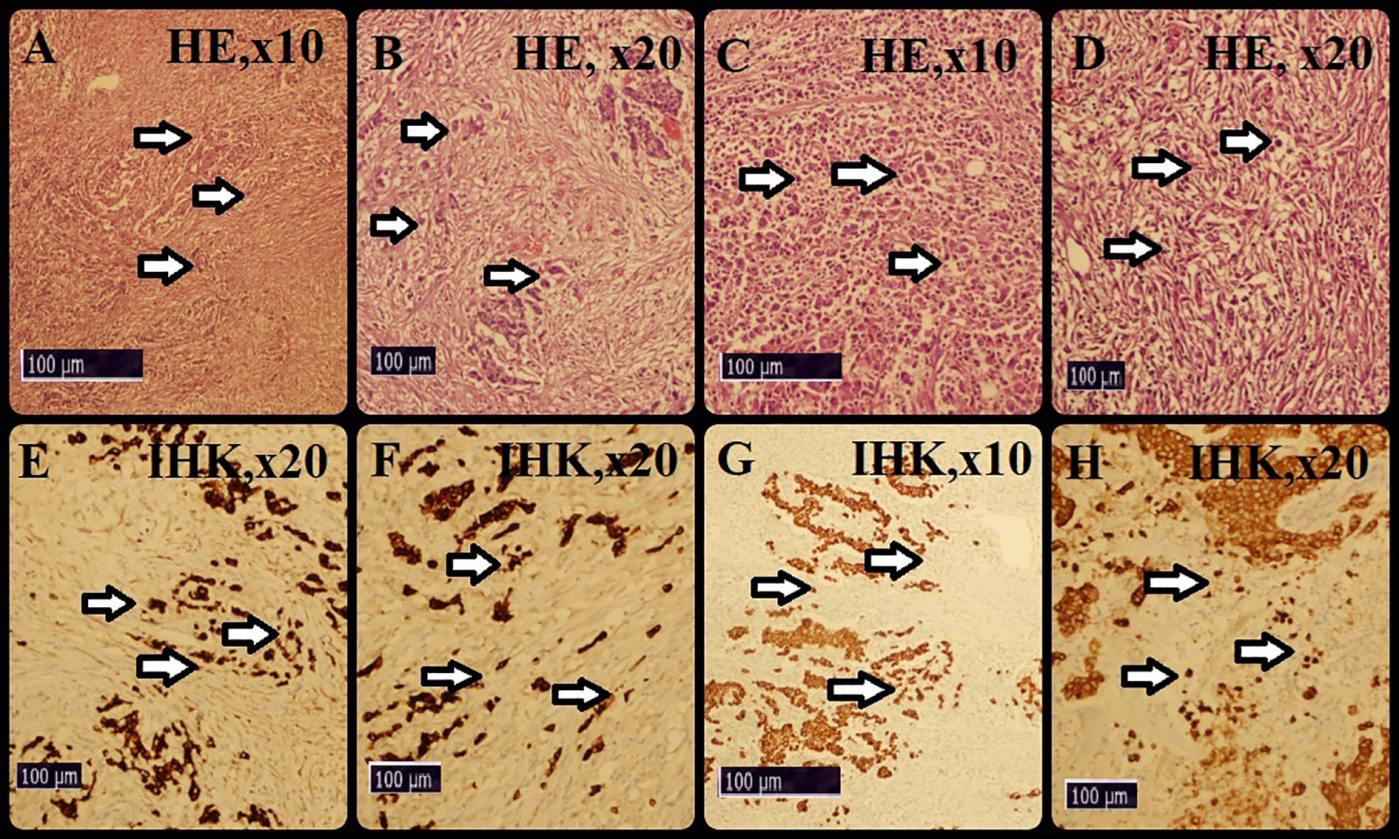
Survival analyses for BD. Survival curves were presented by the Kaplan-Meier test. **(A–D)** Hematoxylin-Eosin samples of BD. **(E–H)** Immunohistochemical samples of BD.

The five-year survival rates showed that RFS was 11% and OS was 14% in patients with high BD, while RFS was 55% and OS was 54% in patients with low BD (**Table 4**). There was a significant difference between survival groups in univariate analysis (RFS <0.001, OS = 0.001). In multivariate analysis, we determined that high BD was an independent poor prognostic factor for locally advanced CRC (RFS: HR = 2.96 [1.48-3.57], p<0.001; OS: HR = 2.69 [1.38-3.70], p <0.001) pT; grade and surgical margin involvement were the other independent parameters (**Table 4**; **Figure 4**).

**Table 4:** Univariate and multivariate survival analysis.

Prognostic factors		Univariable analysis		Multivariable analysis	
		OS	RFS	OS	RFS
		P-value (5-year survival, %)	P-value (5-year survival, %)	P-value (HR 95% CI)	P-value (HR 95% CI)
Age		0.353	0.274	NC	NC
	**<68**	44	45		
	**≥68**	27	27		
Gender		0.481	0.365	NC	NC
	**Female**	43	47		
	**Male**	26	25		
Size		0.556	0.459	NC	NC
	**<6.3**	42	46		
	**≥6.3**	26	25		
Localization		0.499	0.377	NC	NC
	**Right**	43	47		
	**Left**	27	25		
Lymphatic invasion		0.199	0.123	NC	NC
	**No**	48	47		
	**Yes**	24	25		
Perineural invasion		0.533	0.455	NC	NC
	**No**	41	45		
	**Yes**	26	25		
Surgical Margin		0.005*	0.001*	0.009*	0.003*
	**Negative**	50	51	2.36	2.21
	**Positive**	18	15	(1.14-3.36)	(1.36-2.78)
pT		0.0011*	0.003*	0.028*	0.024*
	**pT IV**	51	49	2.21	2.32
	**pT III**	15	13	(1.18-3.33)	(1.42-3.61)
Grade		0.003*	0.001*	0.025*	0.015*
	**Low**	50	51	2.65	2.21
	**High**	14	13	(1.22-3.51)	(1.51-3.58)
Tumor perforation		0.121	0.111	NC	NC
	**Negative**	47	46		
	**Positive**	22	23		
MSI		0.758	0.777	NC	NC
	**MMR-P**	52	48		
	**MMR-D**	26	17		
BD		0.001*	< 0.001*	< 0.001*	< 0.001*
	**Negative**	54	55	2.69	2.96
	**Positive**	14	11	(1.38-3.70)	(1.48-3.57)

*P values of 0.05 and below are significant and are shown in italics.

OS, Overall survival; PT, Pathologic tumor stage; MSI, Microsatellite instable; BD, Tumor budding; MMR-P, Mismatch Repair-Proficient; MMR-P, Mismatch Repair-Deficient.

## 4 Discussion

We investigated the effect of BD on survival in locally advanced CRC patients who had undergone primary colorectal tumor resection without neoadjuvant chemotherapy or radiotherapy. We found that standardizing the methodology is more beneficial and that BD plays an important role in the progression of CRC.

When we examined the literature, we came across variable data regarding the rate and mean of BD (7.11 to 8.05; 19.5% to 45%) (10, 22–25). For example, in one fairly large study, the budding rate and mean were found to be 30% and 7.11, respectively (10). In another study, the mean bud was reported to be 7.95 (23). We think that the source of these differences in the literature is a result of the heterogeneity of the populations and the variability of the methodology. In our study, intratumoral heterogeneity attracted our attention, and, therefore, we evaluated by calculating the average of different BD areas. We think this may change the budding rate and average number of buds. Evaluation methods of BD vary widely in the literature and comprehensive studies are needed to optimize and standardize the assessment.

Although it has been stated in some studies that BD has no prognostic significance (24, 25), a significant part of CRC-related studies indicate that the presence of high BD is associated with shorter survival (8, 10, 15). Our results showed that BD was an independent poor prognostic factor for CRC (both in terms of recurrence and survival). However, it should be remembered that both the patient population and evaluation methods are variable in the studies. In many studies in the literature, BD was evaluated only in routine H&E stained sections and in a single area. Some comprehensive studies have shown that the presence of high BD is an independent survival parameter for CRC (26). To increase homogeneity, we excluded patients treated with early-stage and neoadjuvant chemotherapy. For these reasons, our research was designed to be quite homogeneous compared to other studies.

Although the IHC method is superior in terms of reproducibility and interobserver agreement, a general consensus has been reached regarding the evaluation of BD with H&E (12, 14). Additionally, some studies have yielded similar findings in terms of the prognostic effects of IHC and H&E (11, 24, 27–30, 31). We used a combination of IHC and H&E stained sections, and, as a result, encountered challenges including the presence of the fragmented appearance of tumoral glands due to inflammatory infiltration, the bud-like structures, and the disintegration of tumor tissue due to mucin accumulation. We also had difficulty staining different cell types (for example, vascular cells) in IHC-stained sections.

Significant complications exist in evaluating prognostic parameters such as BD, because researchers use highly variable methods and it is difficult to standardize these dissimilar methods. These techniques include scoring methods, staining, number of areas evaluated, and visualization, among other features (11, 16, 22, 23). In our study, we tried to apply a method that standardized these different methods. The method we used revealed positive results in terms of survival and reproducibility. Therefore, we think that our study contributes to the literature in terms of methodology.

We would like to highlight the important aspects of our study as follows. First, our research was designed in accordance with REMARK guidelines. We studied a highly reliable parameter for CRC patients. Because we studied a homogeneous population with advanced CRC patients who had not received neoadjuvant therapy, our results are quite reliable. We tested a standard methodology and, therefore, our study makes an important contribution to the literature in terms of standardization.

The limitations of our study can be summarized as follows. First, we designed our study retrospectively, and all retrospective studies have some internal limitations (such as sampling bias). Although we tried to evaluate very different areas of the tumor, the proportion of the part we were able to evaluate was quite small when compared to the whole tumor. In addition, since our study included cases before 2005 and treatment was applied according to treatment protocols before that time, it may contain differences according to today’s standards.

## 5 Conclusion

Our results confirm that BD is an independent prognostic factor in CRC patients. We think it is important that BD is present in samples obtained from resection material to predict poor prognosis in locally advanced-stage patients. We also recommend using a standard method to obtain more robust results.

## Data Availability

The original contributions presented in the study are included in the article/supplementary material. Further inquiries can be directed to the corresponding author.
